# AMPK activator O304 improves metabolic and cardiac function, and exercise capacity in aged mice

**DOI:** 10.1038/s42003-021-02837-0

**Published:** 2021-11-18

**Authors:** Madelene Ericsson, Pär Steneberg, Rakel Nyrén, Helena Edlund

**Affiliations:** 1 Umeå Centre for Molecular Medicine Umeå University, SE-901 87 Umeå, Sweden; 2 Department of Medical Biosciences, Pathology Umeå University, SE-901 87 Umeå, Sweden

**Keywords:** Ageing, Type 2 diabetes

## Abstract

Age is associated with progressively impaired, metabolic, cardiac and vascular function, as well as reduced work/exercise capacity, mobility, and hence quality of life. Exercise exhibit positive effects on age-related dysfunctions and diseases. However, for a variety of reasons many aged individuals are unable to engage in regular physical activity, making the development of pharmacological treatments that mimics the beneficial effects of exercise highly desirable. Here we show that the pan-AMPK activator O304, which is well tolerated in humans, prevented and reverted age-associated hyperinsulinemia and insulin resistance, and improved cardiac function and exercise capacity in aged mice. These results provide preclinical evidence that O304 mimics the beneficial effects of exercise. Thus, as an exercise mimetic in clinical development, AMPK activator O304 holds great potential to mitigate metabolic dysfunction, and to improve cardiac function and exercise capacity, and hence quality of life in aged individuals.

## Abstract (web summary)

Ericsson et al. show in aged mice that the panAMPK activator O304 prevents and reverts age-associated hyperinsulinemia and insulin resistance, as well as improving cardiac function and exercise capacity. O304 thus functions as an exercise mimetic and could therefore have a similar therapeutic potential in aged humans and improve quality of life.

## Introduction

Metabolic function, cardiac capacity, and vascular flexibility decline progressively with age, which in combination reduce work capacity, mobility, and quality of life^1^. Type 2 diabetes (T2D) and cardiovascular diseases (CVDs) incidence increase with age and are current global epidemics representing major challenges to health care systems. Regular exercise not only increases work/exercise capacity but also counteracts the development of numerous age-related diseases, including several forms of metabolic and CVDs, thus promoting healthy aging^2–4^. However, aged individuals are frequently unable to engage in regular exercise/physical activity. Thus, there is a large need to develop pharmacological treatment(s) that can increase exercise/work capacity to counteract metabolic dysfunction and improve cardiac and vascular function and thereby promote healthy aging and increase quality of life in the aging population.

Insulin resistance and associated hyperinsulinemia are predictors of age-related diseases such as T2D and CVD^5,6^. Increased activity of AMP-activated protein kinase (AMPK), a key energy sensor that is activated in response to low energy and glucose levels following exercise, enhances insulin-dependent and insulin-independent skeletal glucose uptake, thus improving glucose homeostasis and insulin resistance^7–10^. AMPK activity declines however with age^11^. Thus, AMPK has emerged as a potential important link between, and mediator of, numerous positive effects of exercise including protection against age-related diseases^2,12^.

We previously showed that pan-AMPK activator O304 stimulates AMPK activity and glucose uptake in both skeletal muscle and heart of diet-induced obese (DIO) mice in vivo^10^. In DIO mice, O304 mitigated hyperglycemia, hyperinsulinemia, insulin resistance, and obesity, and in a transgenic type 2 diabetic mouse model, O304 reversed established diabetes^10^. O304 also significantly increased stroke volume, end-diastolic volume, and reduced heart rate in DIO mice^10^, mimicking the cardiac effects of exercise. Thus, AMPK activator O304 efficiently ameliorated obesity-provoked insulin resistance, diabetes, and cardiovascular dysfunction in obese mice. O304 is currently in clinical development, and in a short proof-of-concept phase IIa clinical trial in T2D patients O304 reduced fasting plasma glucose levels and insulin resistance, i.e., HOMA-IR, and increased microvascular perfusion in the calf muscle and reduced blood pressure^10^.

Here we addressed whether AMPK activator O304, in the absence of obesity, can be used to avert insulin resistance, improve cardiac function, and increase exercise capacity in aged mice. Our results show that O304 potently prevents and reverts age-associated insulin resistance and hyperinsulinemia, and additionally improves cardiac function and exercise capacity in lean aged mice. Thus, as an exercise mimetic O304 holds great potential to ameliorate insulin resistance and to improve cardiac and microvascular function, as well as work/exercise capacity, and hence the quality of life in aged individuals.

## Results

### O304 prevents the development of hyperinsulinemia and insulin resistance in aging mice

**Fig. 1:**
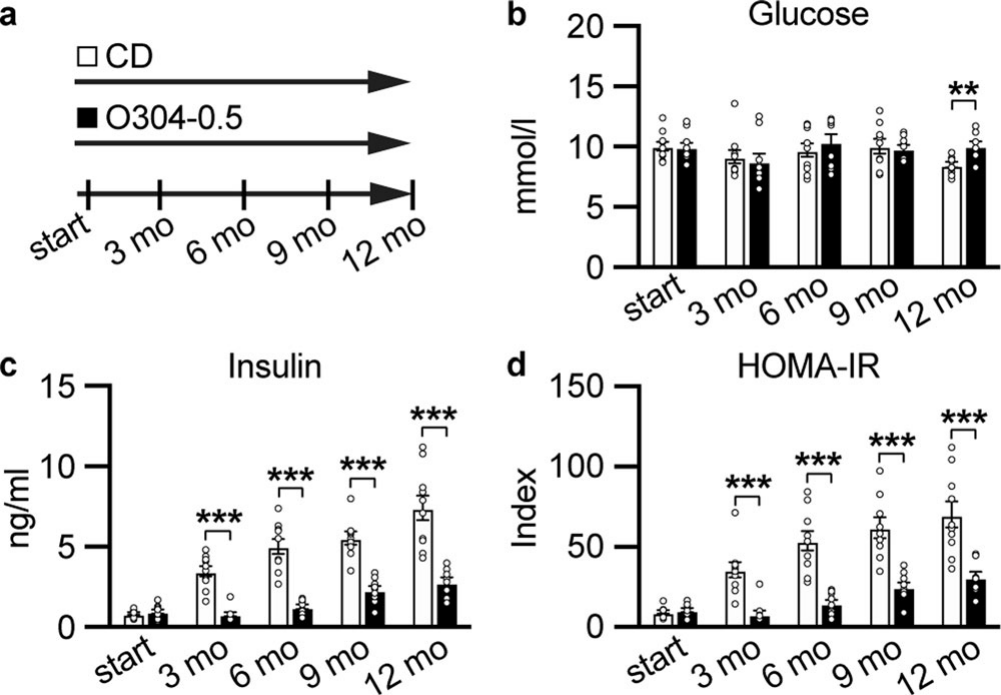
O304 prevents hyperinsulinemia and insulin resistance in aging mice. **a** Timeline in months (mo) for mice fed control diet (CD) or CD supplemented with 0.5 mg/g O304 (O304-0.5) from 6 months of age. **b**–**d** Fasted glucose (**b**), and insulin (**c**), levels as well as HOMA-IR calculations (**d**) (based on glucose and insulin levels in (**b**) and (**c**)), in mice fed CD (*n* = 9–10) or O304-0.5 (*n* = 8–10) for 12 months. Data are presented as mean ± s.d. where **P ≤ 0.01, and ***P ≤ 0.001 between CD and O304-0.5 at the time points indicated in the figure (two-tailed Mann–Whitney test).

To address the effects of the AMPK activator O304 on glucose homeostasis in aging mice, 6-months-old mice CBAxC57BL/6J F1 hybrid mice, hereafter referred to as F1 mice, were fed control diet (CD) or CD formulated with O304, at 0.25 or 0.5 mg/g, denoted O304-0.25 and O304-0.5, respectively for 12 months (Fig. 1a and Supplementary Fig. 1a). Analyses of 6 h fasted blood glucose and insulin levels revealed that, although blood glucose levels did not increase with age in mice on CD, insulin levels increased progressively with age indicating the development of age-related insulin resistance in mice fed CD (Fig. 1b, c and Supplementary Fig. 1b, c). Consequently, HOMA-IR calculations corroborated the development of insulin resistance with age in mice fed CD (Fig. 1d and Supplementary Fig. 1d). Mice on O304-0.25 showed significantly reduced insulin levels after 9 and 12 months of treatment, i.e., at 15 and 18 months of age, compared with that of CD-fed mice (Supplementary Fig. 1c). Consequently, insulin resistance, calculated as HOMA-IR, was significantly reduced at 15 and 18 months of age in mice fed O304-0.25 compared with that of mice fed CD (Supplementary Fig. 1d). In mice fed CD formulated with a higher concentration of O304, O304-0.5, the age-related increase in insulin levels was attenuated already after 3 months of treatment compared with that of mice fed CD (Fig. 1c). Consistently, HOMA-IR was also significantly reduced after 3 months of treatment in mice fed O304-0.5 compared with that of mice on CD (Fig. 1d). Taken together these findings show that O304, dose-dependently, protected mice from developing age-related insulin resistance.

### O304 improves cardiac function in aging mice

**Fig. 2:**
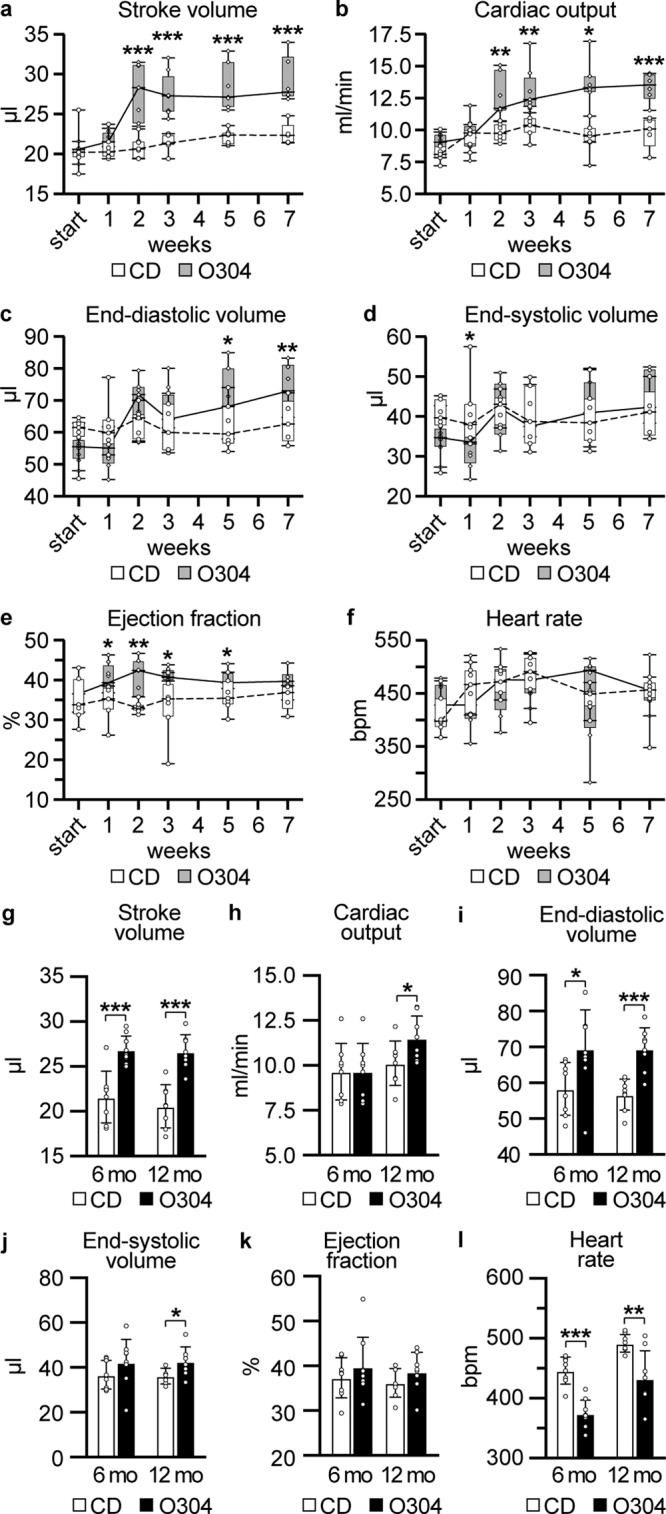
O304 improves cardiac function in aging mice. In all diagrams mice were fed CD or O304-0.5 from 6 months of age. **a**–**f** Stroke volume (**a**), cardiac output (**b**), end-diastolic volume (**c**), end-systolic volume (**d**), ejection fraction (**e**), and heart rate (**f**), in mice fed CD (*n* = 7) or O304-0.5 (*n* = 6) for 7 weeks. **g**–**l** Stroke volume (**g**), cardiac output (**h**), end-diastolic volume (**i**), end-systolic volume (**j**), ejection fraction (**k**), and heart rate (**l**), in mice fed CD (*n* = 8) or O304-0.5 (*n* = 8) for 6 and 12 months (mo). Data are presented as mean ± s.d. Student’s *t*-test between CD and O304-0.5 at respective time point. *P ≤ 0.05, **P ≤ 0.01, and ***P ≤ 0.001.

O304 has been shown to improve cardiac function in DIO mice^10^. To assess whether the protection against the development of hyperinsulinemia and insulin resistance in aging mice was paralleled by improved cardiovascular function in O304 treated mice, cardiac function was assessed by echocardiography. For this purpose, 6-months- old F1 mice were first fed CD or O304-0.5 for 7 weeks. Weekly measurements of cardiac function by echocardiography showed significantly increased stroke volume, end-diastolic volume, and consequently cardiac output, during the first 7 weeks of treatment for O304-0.5 fed mice (Fig. 2a–c). End-systolic volume and heart rate were not changed in mice treated with O304 for 7 weeks, whereas a transient increase in ejection fraction was observed in O304 treated mice (Fig. 2d–f). To explore long-term effects of O304 on cardiac function during aging, echocardiography was performed also after 6 and 12 months of treatment (Fig. 2g–l). Compared with CD-fed mice, stroke volume, cardiac output, and end-diastolic volume, were all significantly increased in O304-0.5 fed mice at 6 and 12 months of treatment (Fig. 2g–i). End-systolic volume tended to be increased after 6 months of O304 treatment and was significantly increased after 12 months of treatment (Fig. 2j), whereas ejection fraction did not differ between O304 treated and CD-fed mice (Fig. 2k). Heart rate was reduced in O304-0.5 fed mice after both 6 and 12 months of treatment (Fig. 2l). Left ventricle wall thicknesses did not differ between CD and O304-0.5 fed mice after the 12 months treatment period (Supplementary Table 1). Taken together, these data show that O304 improved cardiac function in aging mice in an exercise mimicking manner by increasing stroke volume and end-diastolic volume and reducing resting heart rate^13^.

### O304 reduces glucose levels and reverts established hyperinsulinemia and insulin resistance in aged mice

**Fig. 3:**
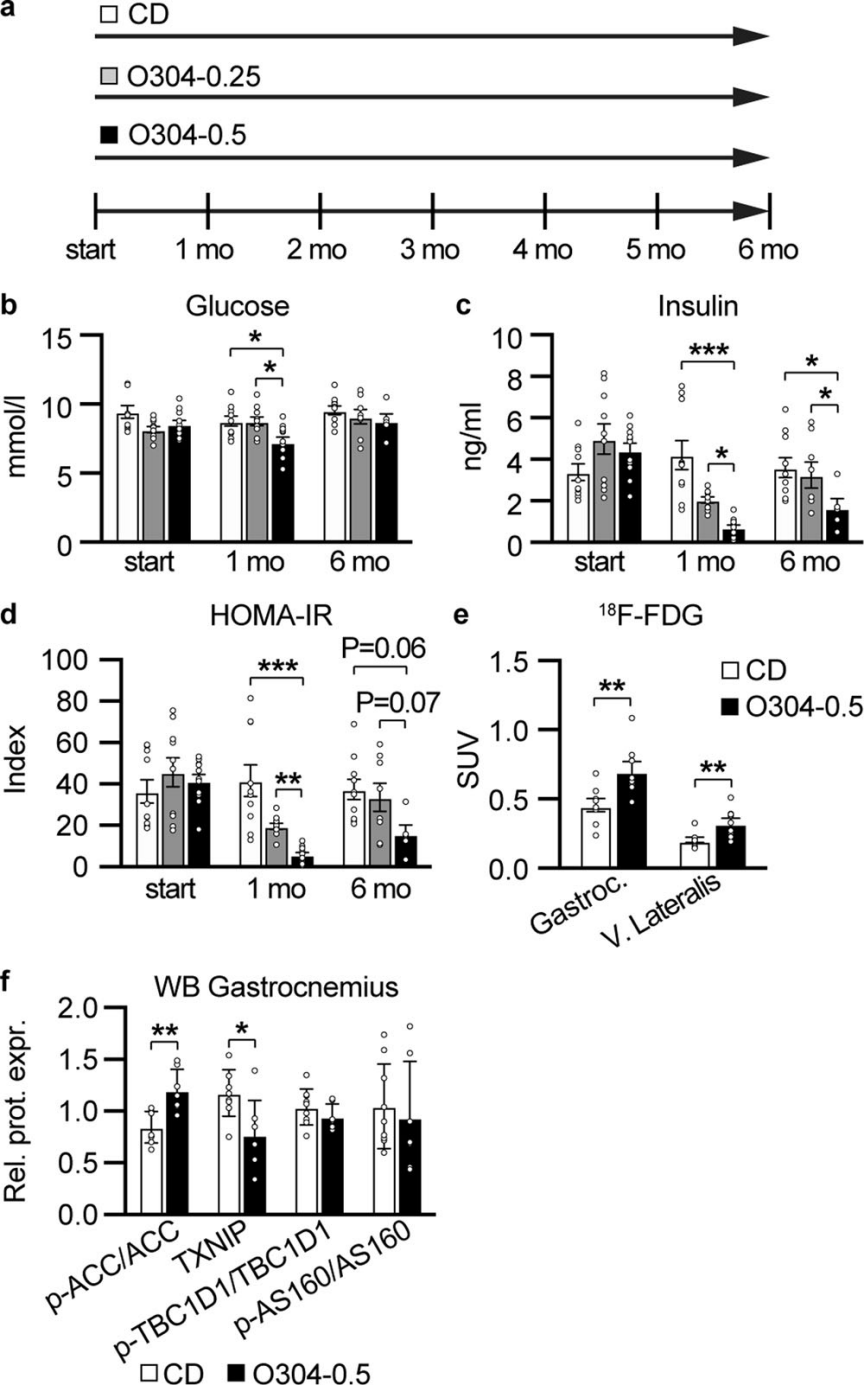
O304 ameliorates hyperinsulinemia and insulin resistance in aged mice. **a** Timeline in months (mo) for mice fed CD or O304-0.25 and O304-0.5, respectively, from 12 months of age. **b**–**d** Fasted glucose (**b**), and insulin (**c**), levels as well as HOMA-IR (**d**) calculations (based on glucose and insulin levels in (**b**) and (**c**)), in mice fed CD (*n* = 9–10), O304-0.25 (*n* = 8–10) or O304-0.5 (*n* = 5–10) before start and at 1 and 6 months of diet. **e** [^18^F]-Fluorodeoxyglucose ([^18^F]-FDG) levels in gastrocnemius and vastus lateralis muscles isolated from mice fed CD (*n* = 8) or O304-0.5 (*n* = 8–9) at 6 months of diet. (**f**), Quantification of p-ACC-Ser ^79^, TXNIP, p-TBC1D1-Ser^231^, and p-AS160, protein levels by western blot analyses of extracts from gastrocnemius muscle in mice fed CD (*n* = 9) or O304-0.5 (*n* = 7) at 6 months of diet. In (**b**–**d**) statistical analyses were performed by one-way ANOVA/Kruskal–Wallis test and in (**e**–**f**) by Student *t*-test. Data are presented as mean±s.d. were *P ≤ 0.05, **P ≤ 0.01, and ***P ≤ 0.001 between CD and O304-0.25 and O304-0.5, respectively, and between O304-0.25 and O304-0.5 at time points indicated in the figure.

Given that O304 appeared to prevent insulin resistance and hyperinsulinemia in aging mice, we next wanted to investigate whether O304 not only could prevent but also ameliorate established age-provoked insulin resistance and hyperinsulinemia. For these purposes, 12-months-old F1 mice were fed CD or O304-0.25 and O304-0.5 for 6 months (Fig. 3a). Mice on O304-0.25 showed evidence of reduced insulin levels and reduced insulin resistance, i.e., HOMA-IR, already after 1 month of treatment compared with that at start and CD-fed mice (Fig. 3b–d). Mice fed O304-0.5 showed even further reductions in insulin and HOMA-IR after the first month of treatment, and additionally had significantly lower glucose levels compared both with that at start and CD-fed mice (Fig. 3b–d). After 6 months of treatment, i.e., at 18 months of age, insulin levels were lower and HOMA-IR was reduced (*P* = 0.06) in mice fed O304-0.5 compared with that of CD-fed mice (Fig. 3b–d). However, insulin and HOMA-IR no longer differed between mice fed CD and mice fed O304-0.25 after 6 months of treatment (Fig. 3b–d). Notably, though, insulin levels tended to increase from 1 to 6 months of treatment in mice fed O304-0.25 (*P* = 0.07), whereas it was unaltered in untreated mice (Fig. 3b–d). These findings may suggest that O304 treated mice partly compensated for insulin resistance by increasing insulin secretion whereas untreated mice failed to do so. Together, these data demonstrate that O304 dose-dependently reduced established, age-related insulin resistance and dysglycemia.

AMPK activation stimulates glucose uptake in skeletal muscle and consequently O304 has been shown to stimulate glucose uptake in skeletal muscle both in vitro and in vivo^10^. To explore whether the O304 mediated reduction of insulin resistance in aged mice involved enhanced muscle glucose uptake, [^18^F]-Fluorodeoxyglucose ([^18^F]-FDG) uptake was assessed by positron emission tomography (PET) scanning in 18-months-old mice that had been fed CD or O304-0.5 for 6 months. Mice fed O304-0.5 showed significantly increased [^18^F]-FDG uptake in gastrocnemius and vastus lateralis muscles compared to mice fed CD (Fig. 3e). Taken together, these findings provide evidence that O304, at least in part, ameliorates age-provoked insulin resistance by enhancing skeletal muscle glucose uptake. The levels of phosphorylated-ACC (Ser 79), a known AMPK target, were increased and the levels of thioredoxin interacting protein (TXNIP), a negative regulator of insulin-dependent and insulin-independent glucose uptake that is targeted for degradation by AMPK1^14–16^, were reduced in skeletal muscles of mice fed O304-0.5 compared with mice fed CD (Fig. 3f and Supplementary Fig. 2). AMPK mediated stimulation of glucose uptake in skeletal muscle in response to exercise has implicated phopshorylation of TBC1D4, also known as Akt substrate (AS160) and TBC1D1^17,18^. We did, however, not find evidence of increased AS160 or TBC1D1-Ser^231^ phosphorylation in skeletal muscles of O304 treated mice (Fig. 3f and Supplementary Fig. 2). Together, these findings provide evidence that O304 promotes glucose uptake in skeletal muscles of aged mice, at least in part, by reducing TXNIP levels.

### O304 improves cardiac function in aged mice

**Fig. 4:**
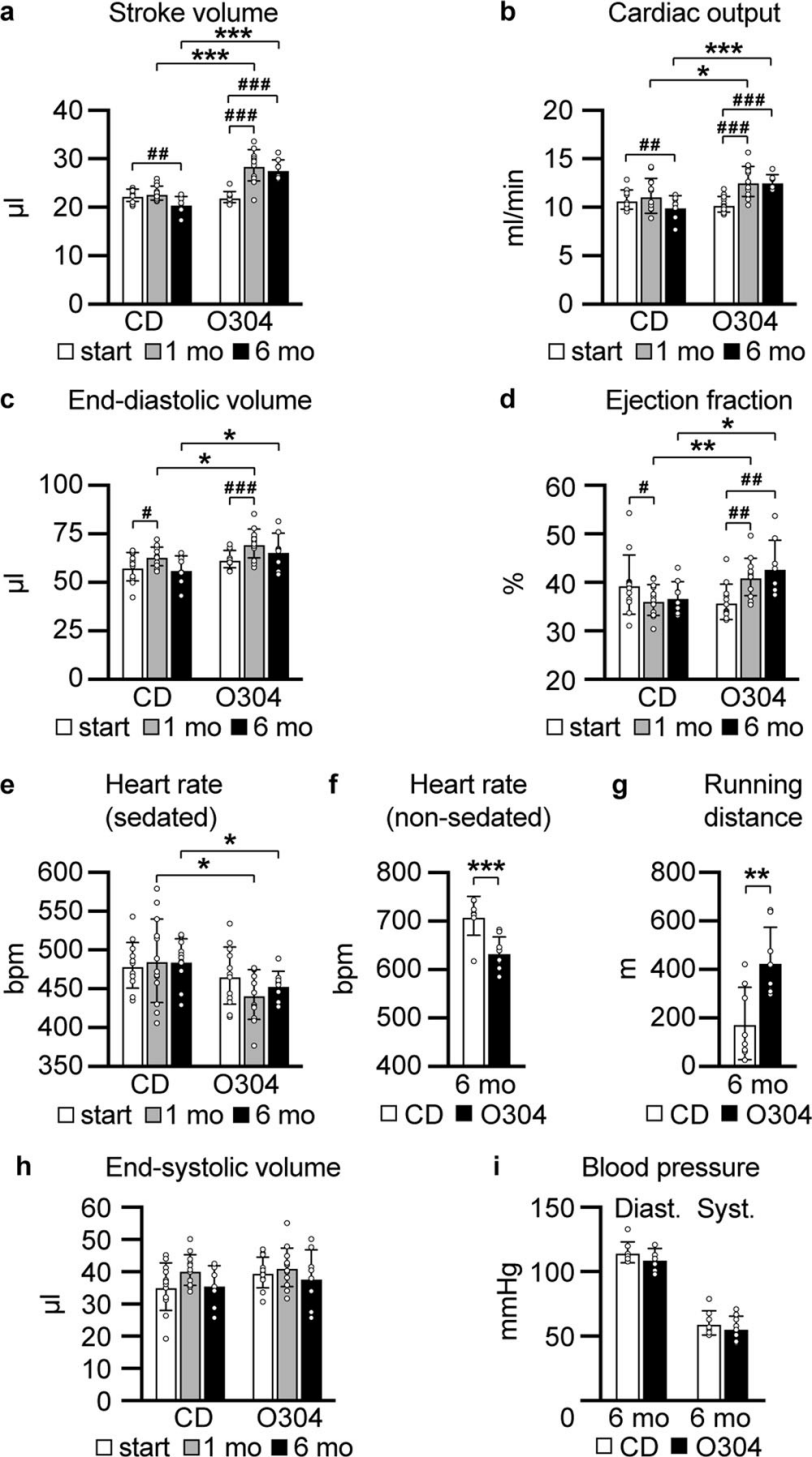
O304 improves cardiac function in aged mice. In all diagrams mice were fed CD or O304-0.5 for 6 months with diet start at 12 months of age. **a**–**e**, **h** Stroke volume (**a**), cardiac output (**b**), end-diastolic volume (**c**), ejection fraction (**d**), heart rate (sedated mice) (**e**), and end-systolic volume (**h**), in mice fed CD (*n* = 7) or O304-0.5 (*n* = 9) before start and at 1 and 6 months (mo) of diet. **f**, **g** Heart rate (non-sedated mice) (**f**), and endurance test (**g**), in mice fed CD (*n* = 8) or O304-0.5 (*n* = 8) at 6 months (mo) of diet. **i** Blood pressure in mice fed CD (*n* = 8) and O304-0.5 (*n* = 8) at 6 months (mo) of diet. Data are presented as mean ± s.d. Student´s *t*-test between CD and O304-0.5 diet groups at respective time point. *P ≤ 0.05, **P ≤ 0.01, and ***P ≤ 0.001.

To elucidate whether O304 not only could ameliorate age-provoked insulin resistance and dysglycemia but also might improve cardiac function in aged mice, we next evaluated cardiac function in 18 months old F1 mice fed CD or O304-0.5 for 6 months. Mice on O304-0.5 showed improved stroke volume, cardiac output, end-diastolic volume, and ejection fraction after 1 and 6 months of treatment compared both with baseline values and with mice fed CD (Fig. 4a–d). Heart rate was reduced in both sedated and non-sedated 18 months old mice treated with O304 for 6 months (Fig. 4e, f), collectively reproducing the results observed in 12 and 18-months-old mice treated with O304 from 6 months of age (Fig. 2l). Consistent with improved cardiac function in O304 treated mice, endurance capacity, monitored as running to exhaustion, was increased in 18-months-old mice treated with O304 for 6 months (Fig. 4g). No differences in end-systolic volume or blood pressure were observed between mice fed CD or O304-0.5 (Fig. 4h, i). Together these data show that in aged mice O304 improved both cardiac function, reminiscent of the cardiac effects of exercise, and endurance capacity.

**Fig. 5:**
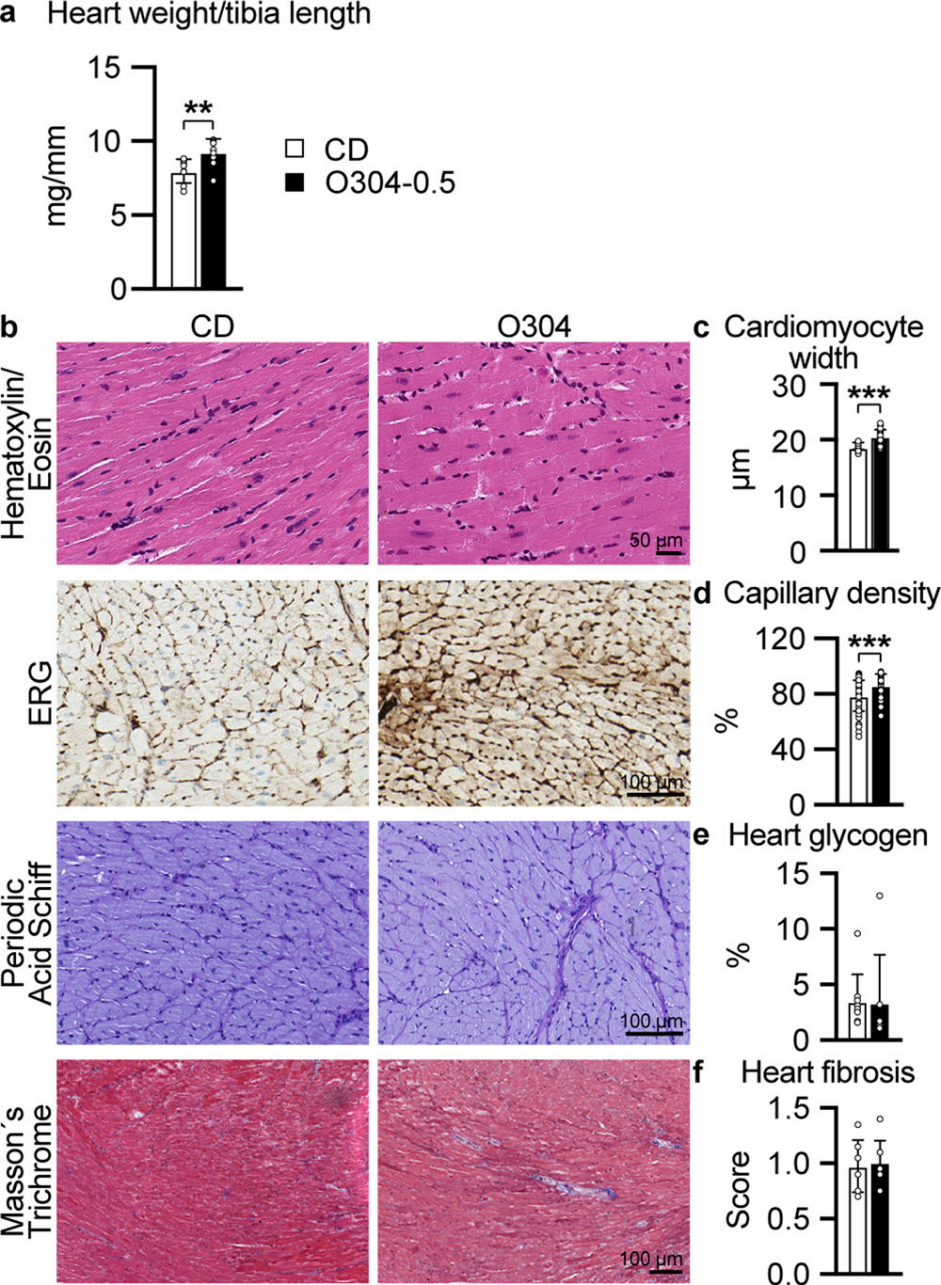
O304 mimics exercise in myocardial tissue by increased cell width and capillary density without increasing fibrosis. All data from 18-months-old mice fed CD or O304-0.5 from 12 months of age. **a** Gravimetric heart weight in mice fed CD (*n* = 8) and O304-0.5 (*n* = 9). **b** Representative images of myocardial sections from mice fed CD and O304-0.5 stained with Hematoxylin and Eosin, ERG antibody representing capillaries, Periodic Acid Schiff and Masson’s trichrome. **c**–**f** Quantification of cardiomyocyte width (**c**), in mice fed CD (*n* = 8) and O304-0.5 (*n* = 8), myocardial capillary density (**d**), in mice fed CD (*n* = 7) and O304-0.5 (*n* = 6), myocardial glycogen content (**e**), in mice fed CD (*n* = 9) and O304-0.5 (*n* = 7) and myocardial fibrotic score (**f**), in mice fed CD (*n* = 8) and O304-0.5 (*n* = 7). Data are presented as mean ± s.d. Student´s *t*-test between CD and O304-0.5 groups. **P ≤ 0.01, and ***P  ≤ 0.001.

AMPK activators have been suggested to function as exercise mimetics^19^, and the increased endurance capacity observed in 18-months-old mice fed O304-0.5 for 6 months supports this notion. Exercise has been shown to be cardioprotective and to be associated with physiological cardiac adaption^20–23^ characterized by mild (10–20%) increase in heart mass^24^. Consistently, although estimated left ventricle wall thicknesses did not differ between 18-months-old mice fed CD and O304-0.5 for 6 months (Supplementary Table 2), heart weight was 15% greater in O304-0.5 fed mice compared with mice fed CD (Fig. 5a). In further agreement with physiological cardiac adaption to exercise, cardiomyocyte width was greater in O304 fed compared to CD-fed mice (Fig. 5b, c). A decline in exercise capacity in old mice is associated with reduced cardiac microvascularization whereas exercise can increase cardiac capillary density^25^. Notably, myocardial capillary density was higher in 18-months-old mice treated with O304 for 6 months (Fig. 5b, d). Increased heart size and cardiac physiological adaption from exercise are not associated with fibrosis or glycogen accumulation^26,27^. Periodic Acid Schiff staining (Fig. 5b), and quantification of glycogen content (Fig. 5e) showed that heart glycogen content was similar in 18-months-old mice fed CD and O304-0.5 for 6 months. Moreover, interstitial fibrosis did not differ between 18-months-old mice fed CD or O304-0.5 for 6 months (Fig. 5b, f). Taken together, these findings show that O304 improved cardiac function in aged mice and that the cardiac effects of O304 largely mimicked the cardiac adaptions to exercise.

## Discussion

With an increasing aging population globally, age-related metabolic and cardiac morbidities represent an increasing health and economic burden to societies worldwide. The development of insulin resistance is not only associated with aging but also a predictor of age-related diseases such as T2D and CVDs. Moreover, cardiac function, work capacity, and mobility decline with age, which reduce the quality of life and are associated with negative health outcomes^4,28^. Exercise is a potent means to prevent and ameliorate insulin resistance and has consequently been shown to positively attenuate age-related diseases^2,3,12,29–32^. Exercise may, however, be challenging and not always a feasible option for aged individuals. Thus, there is an urgent need for therapeutic drugs that can mimic the positive physiological effects of exercise in aged individuals.

Increased AMPK activity has been proposed to extend lifespan in non-vertrebrates^11^. Moreover, AMPK activation exerts several of the positive effects on aging and age-related diseases mediated by caloric restriction and exercise^2,12^. AMPK activity declines however with age^11^, thus pharmacological approaches that can enhance AMPK activity is of great interest. Indirect AMPK activators such as AICAR has been suggested to function as exercise mimetics^2,12^. However, although 4 weeks of AICAR treatment of young, 8-weeks-old mice, increased endurance in a tread-mill test, cardiac function, or metabolic effects associated with aging were not addressed^33^. AICAR also exerts many AMPK-independent effects^34^ and is also not suitable for clinical use. Long-acting direct pan-AMPK activators that interact with the ADaM site has been shown to improve glucose homeostasis in rodents and rhesus monkeys by promoting glucose uptake in skeletal muscle, however, these compounds increase cardiac glycogen and induce substantial cardiac hypertrophy, and have not been proceeded to clinical development^9^. In contrast, structurally related short-acting AMPK activators reduce hyperglycemia and insulin resistance in rodent models without causing increased deposition of cardiac glycogen. However, whereas these compounds induced patterns of gene expression reminiscent of the effects of exercise in several tissues, direct effects on cardiac function or exercise capacity by these compounds were not addressed^35^. Thus, the effects exerted by an AMPK activator on metabolic dysfunction, cardiac function, and exercise capacity in aged mice have to our knowledge not been previously addressed. Here we show that in aged mice, the pan-AMPK activator O304 prevented and reverted age-associated insulin resistance, improved cardiac function, and enhanced exercise capacity in aged mice.

We have previously shown that in DIO mice, O304 prevented and reverted high-fat diet-induced insulin resistance, hyperinsulinemia, obesity, and hepatic steatosis^10^. Our present findings show that O304 prevented and reverted age-provoked insulin resistance and hyperinsulinemia also in lean aging and aged mice. AMPK activation has been suggested to mediate the improved insulin sensitivity evoked by exercise by stimulating both insulin-dependent and insulin-independent skeletal muscle glucose uptake^7–10^. Consistently, skeletal muscle glucose uptake was enhanced in vivo in O304 treated lean aged mice, which likely accounts for the observed positive effect of O304 in preserving and restoring insulin sensitivity in aging and aged mice. Thus, O304 potently averts not only obesity-associated insulin resistance but also age-provoked insulin resistance. Exercise and AMPK both promote glucose uptake via GLUT1 and GLUT4 that reside on the plasma membrane^8,17,18^. Thus, mechanisms regulating the translocation and endocytosis of GLUT1 and GLUT4 ultimately regulate the amount of plasma membrane located GLUT1 and GLUT4, and consequently glucose uptake. TXNIP has been shown to promote GLUT1 and GLUT4 endocytosis and thus negatively regulate glucose uptake^15,16^, and *TXNIP* expression has been reported to be increased in muscles from individuals with impaired glucose tolerance or T2D^14^. Notably, exercise, supposedly in an AMPK dependent manner, has been shown to reduce TXNIP levels in rat skeletal muscle^36^, and AMPK has been shown to phosphorylate TXNIP with subsequent rapid degradation of TXNIP as a result^15^. In addition, AMPK has been shown to phosphorylate and thus inhibit ChREBP, a main transcription factor regulating *TXNIP* expression^37^. Hence, AMPK both directly and indirectly reduces TXNIP levels. Taken together, the reduced expression of TXNIP in muscles of O304 treated mice is consistent with the increased glucose uptake observed in these mice.

TBC1D1 and TBC1D4 are Rab GTPases-activating proteins that have been proposed to mediate exercise, AMPK, and insulin-induced GLUT1 and GLUT4 mobilization, and thus skeletal muscle glucose uptake^17,18^. Insulin-dependent stimulation of glucose uptake and GLUT4 mobilization primarily involves phosphorylation of TBC1D4^38,39^ and interestingly AMPK has been shown to enhance insulin sensitivity in skeletal muscle in a TBC1D4 dependent manner that likely involves phosphorylation of TBC1D4 on Ser^211^ ^38,39^. AMPK mediated stimulation of glucose uptake appears to primarily involve phosphorylation of TBC1D1, which in addition to AKT phosphorylation sites carries several consensus AMPK phosphorylation sites including Ser^231^ ^38,39^. Although we did not observe an increase Ser^231^ phosphorylation of TBC1D1, nor increased p-AS160 phosphorylation, AMPK has been suggested to phosphorylate TBC1D1 at multiple sites, i.e apart from Ser^231^ also at Ser^660^ and Ser^700^ ^40^. Moreover, the p-AS160 antibody used in our study primarily detects Akt mediated phosphorylation rather than a potential AMPK mediated phosphorylation of TBC1D4 at Ser^711^. Thus, although our findings suggest that O304 largely stimulates glucose uptake by reducing TXNIP levels in skeletal muscles of aged mice, we can not fully exclude a contribution from potential phosphorylation of TBC1D1 or TBC1D4.

Moderate physical exercise is associated with an irrefutable improvement in cardiac function and work/exercise capacity, and a reduction in cardiac mortality^13^. A key beneficial change in cardiac function in response to exercise is increased stroke volume, mediated in large part by increased filling of the left ventricle (end-diastolic volume), accompanied by a moderate reduction in heart rate^13^. We previously showed that O304 improved peripheral microvascular perfusion in lean mice and increased endurance capacity in 14 months old mice treated with O304 for 1 month, however, neither metabolic parameters nor cardiac function were addressed in those mice^10^. Our current data show that in 18-months-old mice treated with O304 for 6 and 12 months, respectively, O304 improved cardiac stroke volume, end-diastolic volume, and cardiac output. Moreover, ejection fraction, a measure of the heart´s pumping capacity, tended to be increased in 18-months-old mice treated with O304 for 12 months and was significantly improved in 18-months-old mice treated with O304 for 6 months as compared to age-matched mice on CD. Together these findings provide strong evidence that O304 improves physiological cardiac function in aging and aged mice. In agreement with this notion, 18-months-old mice treated with O304 for 6 months had significantly improved endurance capacity monitored as running distance to exhaustion. During exercise, increased preload leads to increased left ventricle end-diastolic volume and hence increased stroke volume^41^. The increased stroke volume will sustain over some time and to maintain cardiac output during rest, heart rate is reduced^42^. Consistently, heart rate was lower in both sedated and conscious 18-months-old mice treated with O304 for 6 months.

Exercise improvement of cardiac function involves not only increased stroke volume^43^ but also improves myocardial perfusion^23^. We have previously shown that O304 can increase microvascular perfusion in hind paws of DIO mice and in calf muscle of T2D patients^10^. Microvascular rarefaction, i.e., reduced myocardial capillary density, has been reported in aging mice and exercise was shown to ameliorate microvascular rarefaction in old and obese mice, resulting in increased myocardial capillary density compared with that of age-matched sedentary mice^25,44^. Notably, myocardial capillary density was higher in O304-0.5 treated mice compared to untreated mice, suggesting that O304 mimics the effects of exercise also in preventing age-associated microvascular rarefaction. Moreover, the relative increase in myocardial capillary density likely contributes to the improved cardiac function and endurance capacity in O304 treated aged mice. Taken together, the beneficial metabolic, cardiac, and microvascular effects imposed by O304 in aged and obese mice^10^, mirror the key effects of moderately strenuous exercise, thus endorsing O304 as an exercise mimetic.

In response to exercise, improved cardiac function is associated with a mild, 10–20%, physiological increase in heart size without fibrosis or glycogen accumulation^26,27^. In 18-months-old mice treated with O304 for six months a 15% relatively higher heart weight was not associated with enhanced fibrosis or glycogen accumulation, further supporting the exercise mimicking, beneficial, physiological cardiac effects of O304. These results are consistent with previous results that O304 reduced heart glycogen in DIO mice^10^, and that short-acting AMPK activators that interact with the AdAM site reduce hyperglycemia and insulin resistance in rodent models without causing increased deposition of cardiac glycogen^9,35^. Collectively, these results provide evidence that it is the mechanisms of AMPK activation and not AMPK activation per se that can promote cardiac glycogen accumulation^35^.

The metabolic and cardiac effects of O304 in aging mice were only addressed in male mice which may limit the extrapolation of our findings to female mice. However, the use of F1 mice rather than inbred mice reduces the risk that our findings are strain specific. In summary, O304 mimics the beneficial effects of exercise in preventing and reverting insulin resistance, ameliorating age-associated cardiac microvascular rarefaction, and improving key cardiac functions and exercise endurance. Thus, as a clinical stage compound, and by acting as an exercise mimetic, AMPK activator O304 exhibits the potential to not only mitigate T2D and CVDs associated with obesity, but also age-related insulin resistance, declining cardiac function, and physical inactivity/mobility. The preclinical effects of O304 also leave open the possibility of treating other age-related diseases, including heart failure as an advanced form of cardiac impairment associated with severe exercise intolerance.

## Methods

### Mice

Male CBA/CaCrl (#609, Charles River, UK) mice were mated with female C57BL/6J mice (#000664, Jackson Laboratory, US) and F1 male off-spring were used throughout the study. Animals were housed at 12:12 h light/dark cycle in a temperature/humidity controlled (22 °C/50%) room, and ad libitum fed with standard chow (Special Diet Service, CRM(E), 801730, Scanbur, Essex, UK). Two weeks before for study start, diet was changed to CD (D10001, Research diets, Inc, New Brunswick, NJ) for all mice included in the study. At study start, mice either continued on CD, or were fed CD custom formulated by Research Diets with 0.25 mg/g (O304-0.25) or 0.5 mg/g (O304-0.5) O304 (CAS # 1261289-04-6, kindly provided by Betagenon AB, Umeå, Sweden). For fasted metabolic parameters, mice were fasted for 6 h in the morning (07:00-13:00). All experimental procedures were performed on male mice. Animal experiments were approved by the Animal Review Board at the Court of Appeal of Northern Norrland in Umeå (approval numbers A-22-16 and A-29-16) and conducted in accordance with Guidelines for the Care and Use of Laboratory animals.

### Glucose and serum related measurements

Blood glucose was measured using a Glucometer (Accu-chek Aviva, Roche, Sweden) and plasma insulin analyzed via the ultrasensitive mouse insulin ELISA kit (Chrystal Chem Inc. #90080). The homeostasis model for insulin resistance (HOMA-IR) was calculated via: fasting blood glucose (mmol/l) × fasting plasma insulin (μU/ml)/22.5.

### Echocardiographic measurements

Cardiac function was recorded by transthoracic echocardiography (Vevo2100, Fujifilm VisualSonics, Toronto, ON, Canada), using the MS550D transducer, center frequency 40 MHz (Fujifilm VisualSonics, Toronto, ON, Canada), as previously described^45^. Images were collected under light isoflurane anesthesia (Attane VET, VM Pharma, Stockholm, Sweden, 0.8 L/min in O_2_ (g)). By tracing the endocardium in parasternal long-axis view (PLAX), left ventricle end-systolic and end-diastolic volumes were measured off-line, generating stroke volume and cardiac output according to the enclosed software (Vevo LAB 3.2.5, Fujifilm VisualSonics, Toronto, ON, Canada). Mid-ventricular dimensions and wall thicknesses used PLAX M-mode. Mice were kept on a heating plate, and heart rate and respiration rates were continuously supervised. Anesthesia was adjusted to keep the respiration rate above 80 breaths per minute, to avoid severe cardiac-respiratory depression.

### Blood pressure measurements

Blood pressure was analyzed using a BP-2000 blood pressure analysis system (Visitech Systems, Apex, NC). Mice were habituated to the 37 °C platform and specimen holder for one day followed by two days habituation in specimen holder with tails fixed in the air cuff. The experimental measurements were then performed daily for five consecutive days and at the same daily time following Visitech System blood pressure measurement protocol.

### Glucose uptake using PET

Mice were starved for 3 h before being intravenously injected in the tail with 9 ± 1.1 MBq of clinical grade [^18^F]-Fluorodeoxyglucose ([^18^F]-FDG) in saline (prepared at the Nuclear Medicine Department at Norrlands University Hospital, Umeå), in a total volume of 70-100 μL, during light isoflurane anesthesia, as described for echocardiography. Mice were allowed to be awake and freely moving in their cage after injection. After 180 minutes, mice were sacrificed under deep isoflurane anesthesia and blood was removed by retrograde perfusion of 10 ml PBS via the aorta. When the liver was pale, tissues (vastus and gastrocnemius muscles and heart), were collected and scanned ex vivo for a 10 minutes static uptake (nanoScan PET/CT, Mediso, Hungary), to assess uptake in the isolated tissues. Images were reconstructed to a 0.4 × 0.4 mm resolution with a 3D iterative reconstruction with four iterations and four subsets (Mediso Tera-Tomo 3D), covering 98 mm axial distance, employing spike filter, delayed-window random correction, scatter, and CT-based attenuation corrections. Tissue CT-volumes were manually delineated using Hounsfield values, and regions of interest were imported into PET images using imlook4d (www.dicom-port.com). Tracer uptake was quantified as standardized uptake values (SUV), using the formula: SUV = C/(i/m); with C being the measured tissue activity concentration (Bq/mL), i; the injected dose (Bq), and m; the body weight (g). C and i were decay corrected to the same time.

### Treadmill test

A running to exhaustion test was performed as previously described^10^. In short, mice were familiarized with the treadmill by 5 min daily walking sessions three times prior to the test. Running protocol was as follows: 15 min at 18.8 m/min, 5 min at 24.4 m/min, and 27.1 m/min until exhaustion.

### Glycogen determination

Heart glycogen content was determined using a Glycogen Assay Kit (Abcam #ab65620) according to the manufacturer’s recommendations, on snap-frozen tissue.

### Histopathology

General histopathological assessment of left ventricular heart tissue was done with standard hematoxylin and eosin staining using 4 µm formalin-fixed, paraffin-embedded sections. Cardiomyocyte size was measured in proximity to the cell nucleus (*n* = 35) in longitudinal sections by two blinded evaluators and presented as a mean value per individual (*n* = 8 for both CD and O304-0.5 fed mice). Connective tissue collagen was visualized using Masson´s trichrome stain (HT15, Sigma- Aldrich, St Louis, MO, USA) according to the manufacturer’s instructions. A blinded evaluator scored the presence of interstitial fibrosis in twenty randomly selected 0.5 µm^2^ areas per heart. Each area was assessed using a semi-quantitative scoring system (0 = no fibrosis, 1 = slight, 2 = moderate or 3 = intense) and presented as mean value per individual. This method has previously been described by Radovits et al.^26^. Glycogen was visualized using Periodic Acid Schiff staining (87007, Thermo Scientific, Kalamazoo, MI, USA). All histopathological assessments and measurements were made using QuPath version 0.1.2.

### Immunostaining

Formalin-fixed, paraffin-embedded heart tissue was sectioned with a thickness of 4 µm. Capillary density in left ventricle was assessed using a rabbit monoclonal ERG antibody. In short; heat-induced antigen retrieval was performed with Ventana/Roche CC1 antigen retrieval buffer (950-224, Roche Diagnostics, Tucson, AZ, USA). Slides were incubated 1:100 for 32 min with ERG. Positive staining was visualized with ultraViewkit (760-500, Roche Diagnostics, Tucson, AZ, USA). Cardiomyocyte nuclei were counterstained with hematoxylin for 8 min. Endothelial and cardiomyocyte nuclei were quantified in ten randomly selected 0.5 µm^2^ areas per heart using positive cell detection and presented as capillary density (percentage of endothelial cell nuclei/total cell nuclei). Statistics were performed on all randomly selected analyzed areas summarized for each diet treatment group by a two-tailed Student’s *t*-test.

### Western blot analysis

Calf muscles were isolated from non-fasted mice and crushed in a mortar using a pestle and liquid nitrogen and then homogenized in ice cold protein lysis buffer (100 mM Tris pH 6.8, 2% SDS), with protease inhibitor cocktail (Roche #04693124001), 1 tablet/10 ml lysis buffer, and phosphatase inhibitor cocktail (Roche #04693124001), 1 tablet/10 ml lysis buffer. The supernatant was collected after 15 min at 14,000 rpm. Samples were analyzed on 4–15% Criterion^TM^ TGX Stain-Free^TM^ Protein Gels (BIO-RAD #5678084). Primary and secondary antibodies are listed in Supplementary Table 3. Values were normalized to stain-free total protein signal.

### Statistics and reproducibility

Shapiro–Wilk test was used for assuming normal distribution, if assumed normality was not satisfied, Mann–Whitney test was used for two-group comparison, and Kruskal–Wallis when number of groups ≥3 for nonparametric data. Normal distributed data used one-way ANOVA with Tukey’s post hoc test for group wise comparison when number of groups ≥3. Two-tailed Student’s *t*-test was chosen for two-group comparison. IBM SPSS (IBM SPSS Statistics for Windows, Version 26.0. Armonk, NY, US) or GraphPad Prism (version 9.0, GraphPad Software, San Diego, Ca, US) were used for statistical calculations. *p* ≤ 0.05 was considered as statistic significant and levels of significance were denoted as *P ≤ 0.05, **P ≤ 0.01, and ***P ≤ 0.001. Number of animals or independent experiments (n) are indicated in the figure legend. Data, expressed as mean ± standard deviation (s.d.), show high reproducibility between replicate experiments.

### Reporting summary

Further information on research design is available in the Nature Research Reporting Summary linked to this article.

## Supplementary information

Supplementary information

Description of Additional Supplementary Files

Supplementary Data 1

Reporting Summary

## Data Availability

All data generated and/or analyzed during this study are either included in this article (and its Supplementary information and Supplementary Data 1) or are available from the corresponding author on reasonable request.
