# Supplementary material for: AMPK activator O304 improves metabolic and cardiac function, and exercise capacity in aged mice
Source: Commun Biol. 2021 Nov 18;4:1306. doi: 10.1038/s42003-021-02837-0 (PMC8602430; doi:10.1038/s42003-021-02837-0)
Supplement: Supplementary file 1 — Supplementary information [file 42003_2021_2837_MOESM1_ESM.pdf]

Supplementary information

AMPK activator O304 improves metabolic and cardiovascular function, and exercise capacity in aged mice

Madelene Ericsson[1], Pär Steneberg[1], Rakel Nyrén[2], and Helena Edlund[1]

[1]Umeå Centre for Molecular Medicine
Umeå University,
SE-901 87 Umeå, Sweden

[2]Department of Medical Biosciences, Pathology
Umeå University
SE-901 87 Umeå, Sweden

Supplementary figure 1. Glucose and insulin levels and HOMA-IR in mice fed control diet (CD) and O304-0.25 from 6 months of age.

Supplementary figure 2. Western blots

Supplementary table 1. Echocardiographic measurements of mice fed CD or O304-0.5 for 6 and 12 months with diet start at 6 months of age.

Supplementary table 2. Echocardiographic measurements of 12 months old mice at start and after 1 and 6 months fed CD or O304-0.5.

Supplementary table 3. List of antibodies

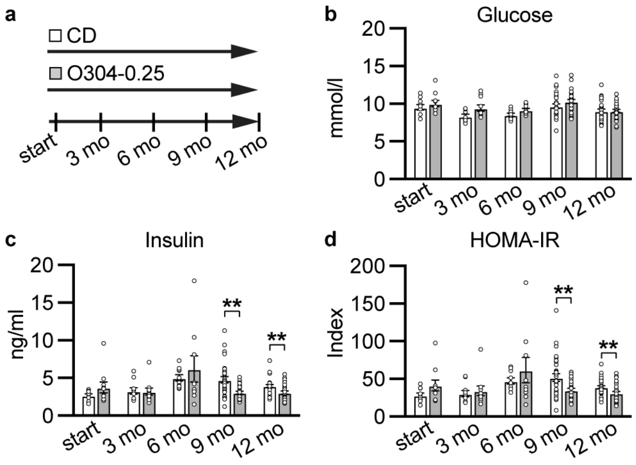

Supplementary figure 1. 0.25 mg/g O304 attenuates development of insulin resistance in aging mice. (**a**) Timeline in months (mo) for mice fed control diet (CD) or CD supplemented with 0.25 mg/g O304 (O304-0.25) from 6 months of age. (**b-d)** Fasted glucose (**b**) and insulin (**c**) levels and HOMA-IR calculations (**d**) (based on glucose and insulin levels in **b** and **c**), in mice fed CD (n=10-30) or O304-0.25 (n=9-27) for 12 months from 6 months of age. Data are presented as mean $\pm$ s.d. where ** $P < 0.01$ between CD and O304-0.25 groups at time points indicated in the figure (two tailed Mann-Whitney test).

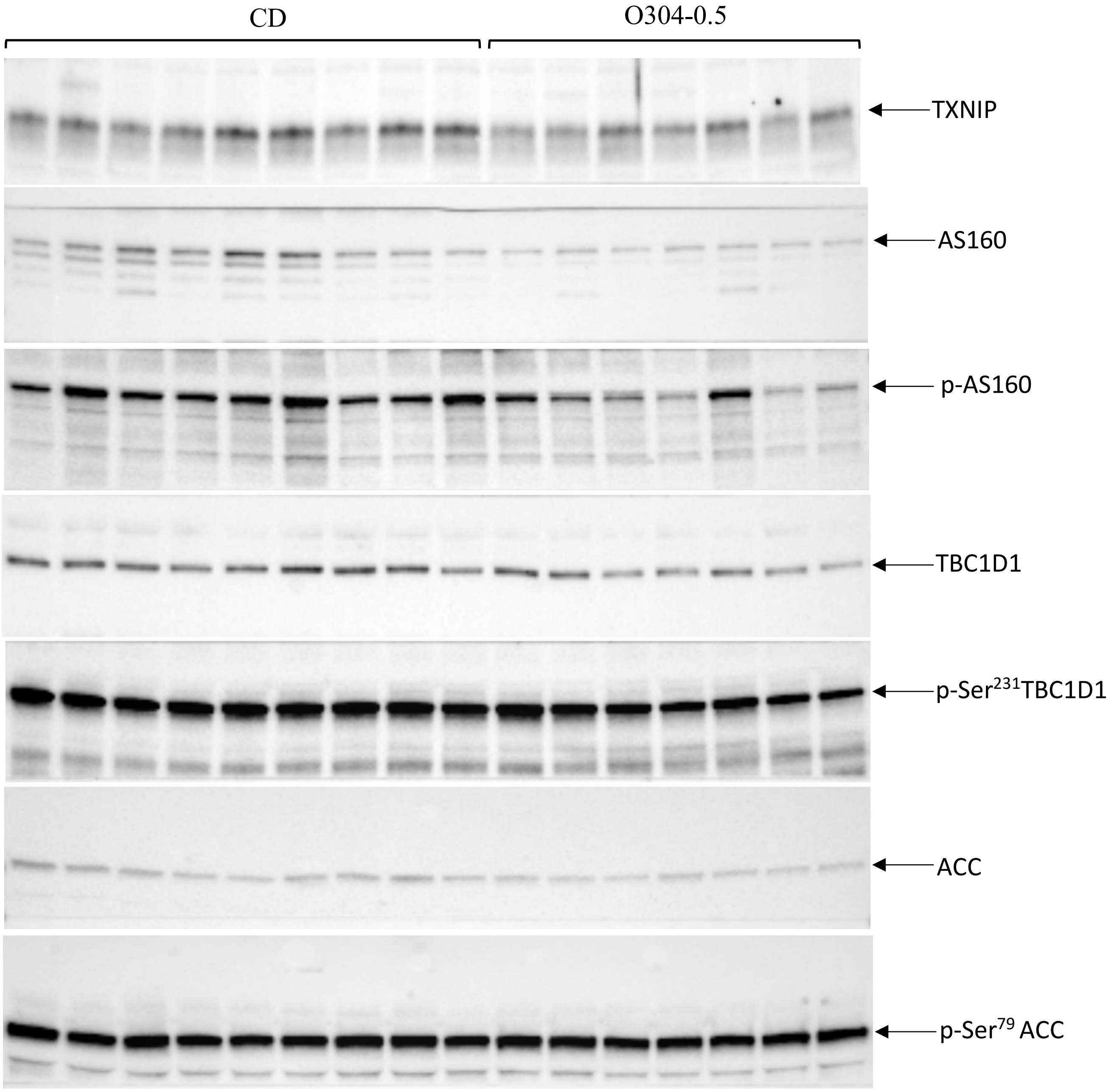

Supplementary figure 2. Western blots of calf muscle from western blot analyses of extracts from gastrocnemius muscle in mice fed CD (n= 9) or O304-0.5 (n=7) at 6 months of diet.

Supplementary table 1. Echocardiographic measurements of mice fed CD or CD supplemented with 0.5 mg/g O304 (O304-0.5) for 6 and 12 months with diet start at 6 months of age.

Group	CD	O304-0.5	CD	O304-0.5
Treatment time	6 months		12 months	
n=	*9*	*9*	*8*	*8*
Body weight (g)	42.6 ± 4.3	41.1 ± 3.1	44.3 ± 3.1	42.1 ± 3.5
B-mode				
HR (bpm)	446 ± 23	374 ± 23***	492 ± 15	433 ± 46**
SV (µL)	21.6 ± 2.89	26.8 ± 1.56***	20.6 ± 2.39	26.6 ± 1.90***
CO (mL/min)	9.7 ± 1.58	10.0 ± 0.70	10.1 ± 1.24	11.5 ± 1.26*
EDV (µL)	58.3 ± 7.31	69.4 ± 10.94*	56.7 ± 4.33	69.4 ± 5.99***
ESV (µL)	36.7 ± 6.39	42.2 ± 10.25	36.2 ± 3.48	42.8 ± 6.44*
EF (%)	37.3 ± 4.49	39.8 ± 6.68	36.2 ± 3.22	38.7 ± 4.33
FS (%)	9.9 ± 2.15	13.0 ± 4.24	10.6 ± 3.56	12.7 ± 3.61
M-mode				
AWd (mm)	0.60 ± 0.06	0.63 ± 0.08	0.59 ± 0.06	0.59 ± 0.06
AWs (mm)	0.77 ± 0.12	0.75 ± 0.06	0.78 ± 0.12	0.77 ± 0.06
LVIDd (mm)	4.55 ± 0.16	4.99 ± 0.13***	4.40 ± 0.27	4.79 ± 0.24*
LVIDs (mm)	3.66 ± 0.10	3.89 ± 0.18**	3.56 ± 0.24	3.89 ± 0.27*
PWd (mm)	0.80 ± 0.08	0.75 ± 0.14	0.87 ± 0.13	0.85 ± 0.15
PWs (mm)	0.98 ± 0.09	0.90 ± 0.13	1.09 ± 0.17	1.08 ± 0.16

Parasternal long-axis B-mode measurements of left ventricular function. HR, heart rate; SV, stroke volume; CO, cardiac output; EDV, end-diastolic volume; ESV, end-systolic volume; EF, ejection fraction and FS, fractional shortening.

Parasternal long-axis M-mode measurements of left ventricular dimensions. AWd/s, anterior wall thickness in diastole/systole; LVIDd/s, left ventricular inner diameter in diastole/systole and PWd/s, posterior wall thickness diastole/systole.

Data are presented as mean ± s.d. P-values are based on Student´s t-test between CD and O304-0.5 groups at respective time point. * $P < 0.05$, ** $P < 0.01$ and *** $P < 0.001$.

Supplementary table 2. Echocardiographic measurements 12 months old mice at start and after 1 and 6 months fed CD or O304-0.5.

Treatment time	0		1 month		6 months	
Group	CD	O304-0.5	CD	O304-0.5	CD	O304-0.5
n	*13*	*13*	*13*	*13*	*11*	*9*
Body weight (g)	43.0 ± 3.4	44.4 ± 4.3	43.1 ± 1.9	39.2 ± 3.5	44.5 ± 2.7	41.6 ± 4.5
B-mode						
HR (bpm)	480 ± 28	467 ± 35	486 ± 52	443 ± 31*	486 ± 27	455 ± 17*
SV (µL)	22.5 ± 1.21	22.1 ± 1.11	22.9 ± 1.38	28.7 ± 3.06***###	20.7 ± 1.44##	27.8 ± 1.85***###
CO (mL/min)	10.8 ± 1.13	10.3 ± 0.77	11.2 ± 1.73	12.7 ± 1.48*###	10.1 ± 1.05	12.6 ± 0.67***###
FS (%)	10.2 ± 3.6	9.8 ± 2.25	9.8 ± 2.56	12.6 ± 2.20**##	10.3 ± 2.10	11.4 ± 3.50
EF (%)	39.6 ± 6.5	36.0 ± 3.46	36.3 ± 3.06#	41.1 ± 3.68**##	36.9 ± 3.05	42.9 ± 5.42*##
EDV (µL)	58.0 ± 7.6	62.0 ± 4.19	63.4 ± 4.6#	70.1 ± 7.19*##	56.7 ± 6.61	65.9 ± 8.83*
ESV (µL)	35.4 ± 7.7	39.8 ± 4.55	40.5 ± 4.59	41.4 ± 5.78	35.9 ± 5.63	38.1 ± 8.16
M-mode						
AWd (mm)	0.53 ± 0.03	0.50 ± 0.02	0.56 ± 0.05	0.53 ± 0.03#	0.55 ± 0.03	0.54 ± 0.04#
AWs (mm)	0.63 ± 0.04	0.64 ± 0.05	0.67 ± 0.05#	0.66 ± 0.07	0.68 ± 0.04#	0.65 ± 0.06
LVIDd (mm)	4.70 ± 0.22	4.80 ± 0.15	4.76 ± 0.16	5.11 ± 0.27**##	4.62 ± 0.27	4.96 ± 0.41
LVIDs (mm)	3.63 ± 0.20	3.72 ± 0.09	3.63 ± 0.29	3.73 ± 0.27	3.44 ± 0.23	3.66 ± 0.40
PWd (mm)	0.75 ± 0.07	0.75 ± 0.05	0.80 ± 0.08	0.74 ± 0.09	0.81 ± 0.11	0.80 ± 0.11
PWs (mm)	0.92 ± 0.04	0.95 ± 0.07	0.96 ± 0.05#	0.91 ±0.10	1.01 ± 0.08##	1.00 ± 0.12

Parasternal long-axis B-mode measurements of left ventricular function. HR, heart rate; SV, stroke volume; CO, cardiac output; EDV, end-diastolic volume; ESV, end-systolic volume; EF, ejection fraction and FS, fractional shortening.
Parasternal long-axis M-mode measurements of left ventricular dimensions. AWd/s, anterior wall thickness in diastole/systole; LVIDd/s, left ventricular inner diameter in diastole/systole and PWd/s, posterior wall thickness diastole/systole.
Data are presented as mean ± s.d. P-values are based on Student´s t-test between CD and O304-0.5 groups at respective time point where * $P < 0.05$, ** $P < 0.01$ and *** $P < 0.001$ or within groups compared to start of treatment $^{\#}p<0.05$, $^{\#\#}p<0.01$, $^{\#\#\#}p<0.001$.

Supplementary table 3. Antibodies

	Antigen	Species	Supplier	Dilution
Primary antibodies	p-ACC (Ser-79)	Rabbit	Cell signaling (cat. nr. 3661)	1:1000
	panACC	Rabbit	Cell signaling (cat. nr. 3662)	1:1000
	p-AS160	Rabbit	Cell signaling (cat. nr. 9611)	1:1000
	AS160	Rabbit	Cell signaling (cat. nr. 2670)	1:1000
	p-TBC1D1 (Ser-231)	Rabbit	MerckMillipore (cat. nr. 072268)	1:500
	TBC1D1	Rabbit	Cell signaling (cat. nr. 4629)	1:1000
	TXNIP	Rabbit	Abcam (cat.nr. 188865)	1:2000
	ERG	Rabbit	Cell Marque (cat.nr. 434R)	1:100
secondary antibodies	peroxidase-conjugated Affini-pure Goat Anti-Rabbit IgG (H+L)		Jackson Laboratories, INC. (cat.nr. 111-035-003)	1:10000
