# Supplementary material for: AMPK activator O304 improves metabolic and cardiac function, and exercise capacity in aged mice
Source: Commun Biol. 2021 Nov 18;4:1306. doi: 10.1038/s42003-021-02837-0 (PMC8602430; doi:10.1038/s42003-021-02837-0)
Supplement: Supplementary file 2 — Description of Additional Supplementary Files [file 42003_2021_2837_MOESM2_ESM.pdf]

Description of Additional Supplementary Files

File name: Supplementary Data 1.

Description: Source data for: Fig 1b fasted glucose levels, Fig 1c fasted insulin levels, Fig 1d HOMA-IR, Fig 2a stroke volume, Fig 2b cardiac output, Fig 2c end-diastolic volume, Fig 2d endsystolic volume, Fig 2e ejection fraction, Fig 2f heart rate, Fig 2g stroke volume, Fig 2h cardiax output, Fig 2i end-diastolic volume, Fig 2j end-systolic volume, Fig 2k ejection fraction, Fig 2l heart rate, Fig 3b fasted glucose levels, Fig 3c fasted insulin levels, Fig 3d HOMA-IR, Fig 3e standard uptake value, Fig 3f relative protein expression, Fig 4a stroke volume, Fig 4b cardiac output, Fig 4c end-diastolic volume, Fig 4d ejection fraction, Fig 4e heart rate, Fig 4f heart rate nonsedated, Fig 4g treadmill running distance, Fig 4h end-systolic volume, Fig 4i blood pressure, Fig 5a heart weight/tibia length, Fig 5c cardiomyocyte width, Fig 5d capillary density, Fig 5e heart glycogen, Fig 5f heart fibrosis score.
