# Supplementary material for: AMPK activator O304 improves metabolic and cardiac function, and exercise capacity in aged mice
Source: Commun Biol. 2021 Nov 18;4:1306. doi: 10.1038/s42003-021-02837-0 (PMC8602430; doi:10.1038/s42003-021-02837-0)
Supplement: Supplementary file 4 — Reporting Summary [file 42003_2021_2837_MOESM4_ESM.pdf]

nature portfolio

Corresponding author(s): Helena Edlund
Last updated by author(s): Oct 14, 2021

Reporting Summary

Nature Portfolio wishes to improve the reproducibility of the work that we publish. This form provides structure for consistency and transparency in reporting. For further information on Nature Portfolio policies, see our Editorial Policies and the Editorial Policy Checklist.

Statistics

For all statistical analyses, confirm that the following items are present in the figure legend, table legend, main text, or Methods section.

n/a	Confirmed	
☐	☒	The exact sample size (n) for each experimental group/condition, given as a discrete number and unit of measurement
☐	☒	A statement on whether measurements were taken from distinct samples or whether the same sample was measured repeatedly
☐	☒	The statistical test(s) used AND whether they are one- or two-sided<br>*Only common tests should be described solely by name; describe more complex techniques in the Methods section.*
☒	☐	A description of all covariates tested
☐	☒	A description of any assumptions or corrections, such as tests of normality and adjustment for multiple comparisons
☐	☒	A full description of the statistical parameters including central tendency (e.g. means) or other basic estimates (e.g. regression coefficient) AND variation (e.g. standard deviation) or associated estimates of uncertainty (e.g. confidence intervals)
☒	☐	For null hypothesis testing, the test statistic (e.g. F, t, r) with confidence intervals, effect sizes, degrees of freedom and P value noted<br>*Give P values as exact values whenever suitable.*
☒	☐	For Bayesian analysis, information on the choice of priors and Markov chain Monte Carlo settings
☒	☐	For hierarchical and complex designs, identification of the appropriate level for tests and full reporting of outcomes
☒	☐	Estimates of effect sizes (e.g. Cohen's d, Pearson's r), indicating how they were calculated

Our web collection on statistics for biologists contains articles on many of the points above.

Software and code

Policy information about availability of computer code

Data collection	Echocardiography used VisualSonics Fujifilm, Vevo2100 (https://www.visualsonics.com/)<br>PET-CT was aquired by nanoScan PET/CT (Mediso, Hungary, https://mediso.com/global/en/product/pre-clinical-products/nanoscanr-petct)<br>Blood pressure measurement on non-sedated mice was aquired by BP-2000 Visitech
Data analysis	Histopathology analyses used QuPath version 0.1.2 (https://qupath.github.io/)<br>Echocardiography analyses used VevoLab v.3.2.5 (VisualSonics Fujifilm https://www.visualsonics.com/product/software/vevo-lab)<br>PET data used imlook4d (www.dicom-port.com)<br>Echocardiography was statistically evaluated using SPSS v26.0 (https://www.ibm.com/products/spss-statistics)<br>GraphPad Prism v 9.0 was used for all other statistical evaluations and comparisons (https://www.graphpad.com/scientific-software/prism/)

For manuscripts utilizing custom algorithms or software that are central to the research but not yet described in published literature, software must be made available to editors and reviewers. We strongly encourage code deposition in a community repository (e.g. GitHub). See the Nature Portfolio guidelines for submitting code & software for further information.

March 2021

Data

Policy information about availability of data

All manuscripts must include a data availability statement. This statement should provide the following information, where applicable:

- Accession codes, unique identifiers, or web links for publicly available datasets
- A description of any restrictions on data availability
- For clinical datasets or third party data, please ensure that the statement adheres to our policy

All data generated and/or analysed during this study are either included in this article (and its Supplementary information) or are available from the corresponding author on reasonable request.

Field-specific reporting

Please select the one below that is the best fit for your research. If you are not sure, read the appropriate sections before making your selection.

☒ Life sciences ☐ Behavioural & social sciences ☐ Ecological, evolutionary & environmental sciences

For a reference copy of the document with all sections, see nature.com/documents/nr-reporting-summary-flat.pdf

Life sciences study design

All studies must disclose on these points even when the disclosure is negative.

Sample size	No sample size calculations were performed. The sample size (n) of each experiment is provided in the corresponding figure captions in the main manuscript and supplementary information files. Sample sizes were chosen to support meaningful conclusions.
Data exclusions	Mice with apparent health problems, such as tumor development, >10% reduction in body weight, or fighting were excluded with no differences between groups.
Replication	All in vivo experiments were successfully replicated 2-3 times. Time-staggered cohorts of F1 male off-spring were randomly housed in groups of 5 mice/cage. Mice were housed until 6 or 12 months of age before each diet treatment was spread evenly across the cohorts ensuring no bias effect of cohort or batch of mice.<br>In vitro analysis of tissues were successfully repeated 2-3 times between different cohorts.
Randomization	In the different time-staggered cohorts, mice were randomly allocated to the cages and different treatments to ensure randomization of cohort and diet effects. Mice from each diet group were randomly selected for in vivo analyses such as echocardiography, PET-scanning, blood pressure, treadmill running and metabolic measurements.<br>For in vitro analyses such as western blot, histology and immuno-histology work samples from 6-9 mice/diet were randomly selected.
Blinding	Cardiomyocyte size was examined by two blinded evaluators.<br>Histological staininings for cardiac fibrosis and glycogen was examined by one blinded evaluator.<br>Echocardiography analyses was performed by one blinded evaluator.<br>Blood pressure measurements and treadmill running was performed by one blinded performer.

Reporting for specific materials, systems and methods

We require information from authors about some types of materials, experimental systems and methods used in many studies. Here, indicate whether each material, system or method listed is relevant to your study. If you are not sure if a list item applies to your research, read the appropriate section before selecting a response.

Materials & experimental systems

n/a	Involved in the study	
☐	☒	Antibodies
☒	☐	Eukaryotic cell lines
☒	☐	Palaeontology and archaeology
☐	☒	Animals and other organisms
☒	☐	Human research participants
☒	☐	Clinical data
☒	☐	Dual use research of concern

Methods

n/a	Involved in the study	
☒	☐	ChIP-seq
☒	☐	Flow cytometry
☒	☐	MRI-based neuroimaging

Antibodies

Antibodies used	ERG Rabbit Cell Marque (cat.nr. 434R)<br>TXNIP Rabbit Abcam (cat.nr. 188865)<br>TBC1D1 Rabbit Cell signaling (cat. nr. 4629)<br>p-TBC1D1 (Ser-231) Rabbit MerckMillipore (cat. nr. 072268)<br>AS160 Rabbit Cell signaling (cat. nr. 2670)<br>p-AS160 Rabbit Cell signaling (cat. nr. 9611)<br>ACC Rabbit Cell signaling (cat. nr. 3662)<br>p-ACC (Ser-79) Rabbit Cell signaling (cat. nr. 3661<br><br>secondary antibodies peroxidase-conjugated Affini-pure Goat Anti-Rabbit IgG (H+L) Jackson Laboratories, INC. (cat.nr. 111-035-003)
Validation	ERG (https://www.cellmarque.com/antibodies/CM/2195/ERG_EP111)

Animals and other organisms

Policy information about studies involving animals; ARRIVE guidelines recommended for reporting animal research

Laboratory animals	F1 male off-spring from crossing of male CBA/CaCrl (#609, Charles River, UK) and female C57BL/6J (#000664, Jackson Laboratory, US) were used throughout the study.
Wild animals	No wild animals were involved in this study.
Field-collected samples	No field-collected samples were collected for this study.
Ethics oversight	Animal experiments were approved by the Animal Review Board at the Court of Appeal of Northern Norrland in Umeå and conducted in accordance with Guidelines for the Care and Use of Laboratory animals.

Note that full information on the approval of the study protocol must also be provided in the manuscript.
